# Supplementary figures and images for: Can flavoprotein monooxygenases functionalize long-chain n-alkanes?
Source: PLoS One. 2025 Sep 19;20(9):e0332702. doi: 10.1371/journal.pone.0332702 (PMC12449030; doi:10.1371/journal.pone.0332702)

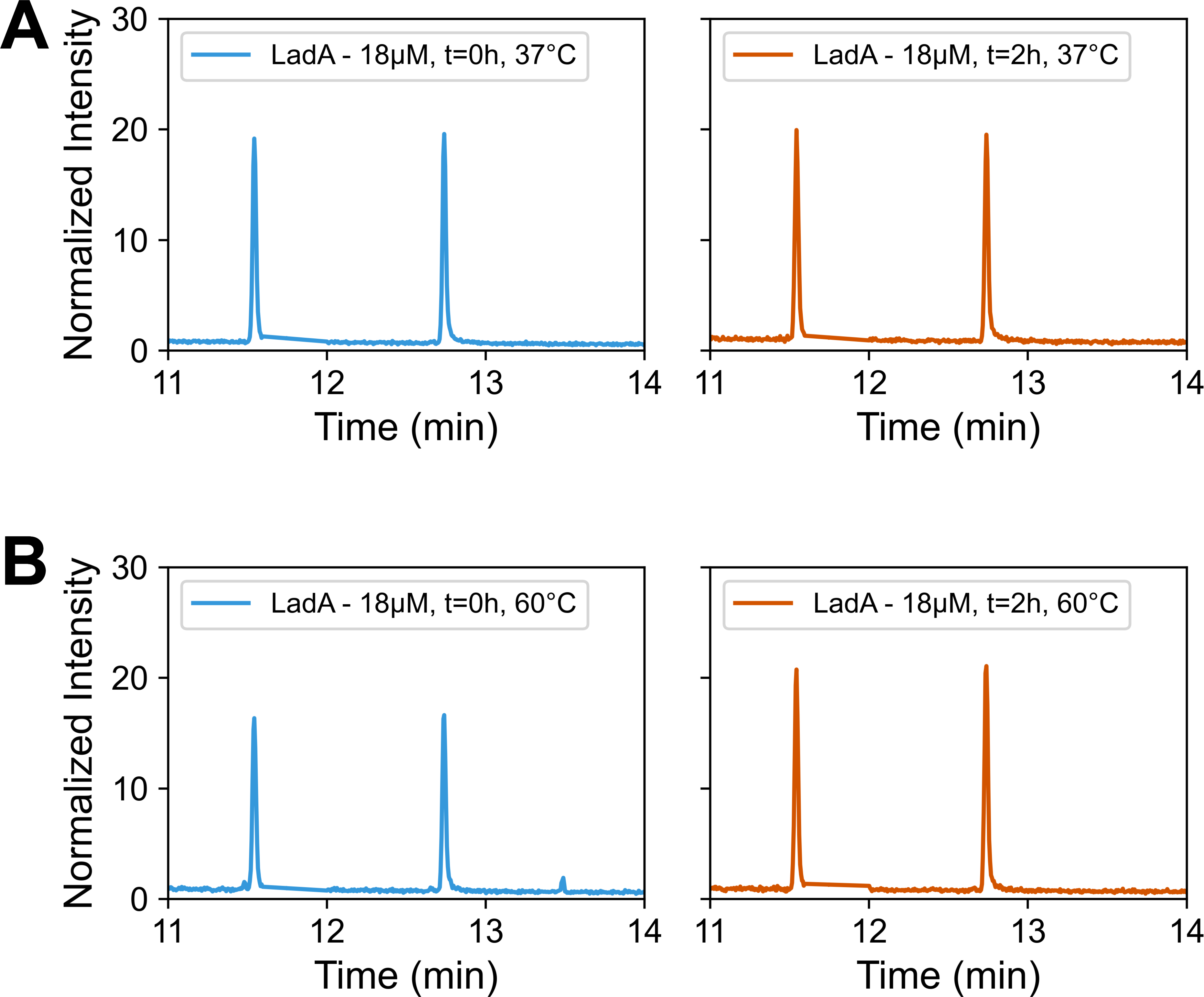

Supplement: S1 Fig — Assayed reaction conditions are as follows: (A) Wild-type N-Strep-tag® LadA (18 μM), hexadecane (1 mM, tR = 12.74 min), FMN (1 mM), NADH (1 mM), MgCl2 (1mM) and HITENOL-AR 0.5% (m/v) for 2 hours at 37°C. (B) N-Strep-tag® LadA:P5 (18 μM), hexadecane (1 mM, tR = 12.74 min), FMN (1 mM), NADH (1 mM) and HITENOL-AR 0.5% (m/v) for 2 hours at 37°C. (TIF) [file pone.0332702.s001.tif]

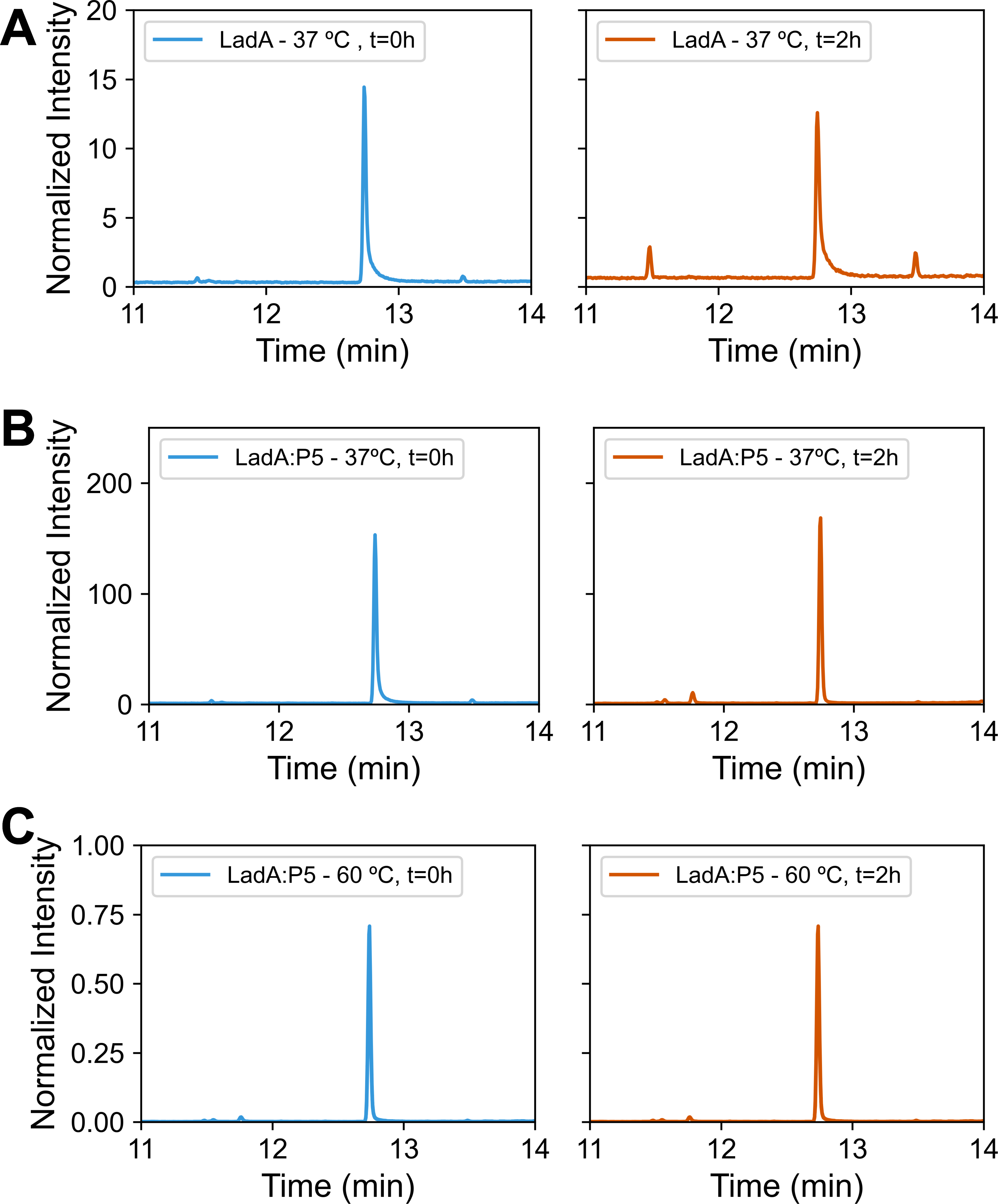

Supplement: S2 Fig — Assayed reaction conditions are as follows: (A) Wild-type N-Strep-tag® LadA (18 μM), wild-type N-Strep-tag® Fre (6 μM), hexadecane (1 mM, tR = 12.74 min), FMN (1 mM), NADH (1 mM) and HITENOL-AR 0.5% (m/v) for 2 hours at 37°C. (B) N-Strep-tag® LadA:P5 (18 μM), wild-type N-Strep-tag® Fre (6 μM), hexadecane (1 mM, tR = 12.74 min), FMN (1 mM), NADH (1 mM) and HITENOL-AR 0.5% (m/v) for 2 hours at 37°C. (C) N-Strep-tag® LadA:P5 (18 μM), wild-type N-strepTag Fre (6 μM), hexadecane (1 mM, tR = 12.74 min), FMN (1 mM), NADH (1 mM) and HITENOL-AR 0.5% (m/v) for 2 hours at 60°C. Additional peak at tR = 11.54 corresponds to pentadecane (internal standard). (TIF) [file pone.0332702.s002.tif]

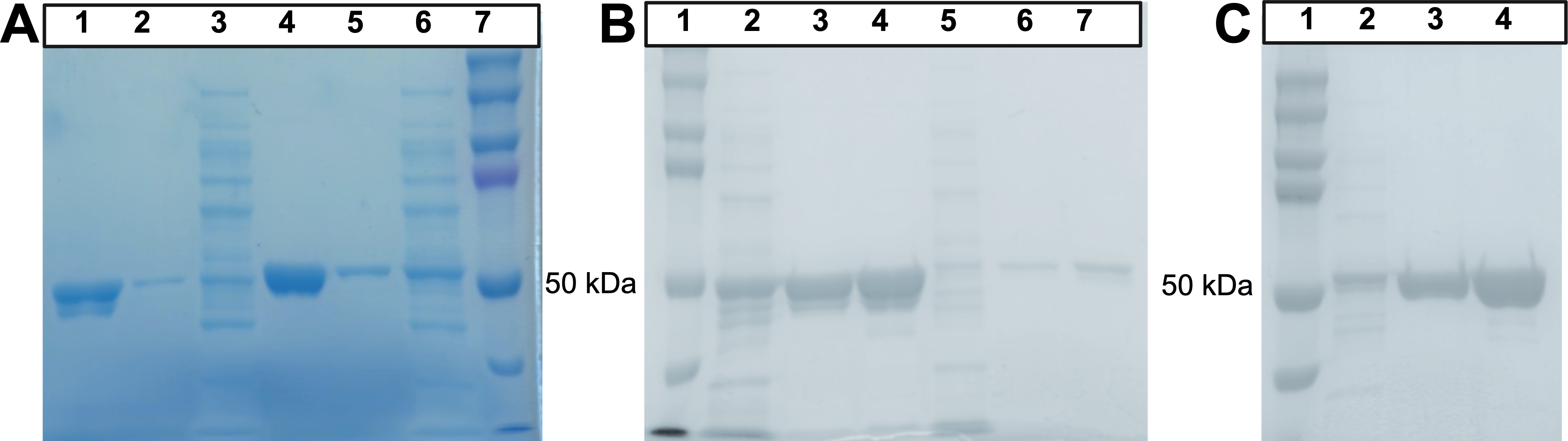

Supplement: S3 Fig — (A) SDS-PAGE for Pseudomonas sp. ANT H4 (WP_149412683.1) and P. viridiflava p8B7 (WP_122428611.1) expression and purification. Lane 1 and 2 purified fractions of Pseudomonas sp. ANT H4. Lane 3 – Crude Lysate for Pseudomonas sp. ANT H4. Lane 4 and 5 – purified fractions of P. viridiflava p8B7. Lane 6 – Crude Lysate for P. viridiflava p8B7. Lane 7 – Molecular weight marker. (B) SDS-PAGE for Mesorhizobium sp. (WP_023806228.1) and P. viridiflava KF4851 (WP_088236120.1) expression and purification. Lane 1 – Molecular weight marker. Lane 2 – Crude Lysate for Mesorhizobium sp. expression. Lane 3 and 4 – purified fractions of Mesorhizobium sp. Lane 5 – Crude Lysate for P. viridiflava KF4851 Lane 6 and 7 – purified fractions of P. viridiflava KF4851 (C) SDS-PAGE for A. rhizogenes (WP_174015212.1) expression and purification. Lane 1 – Molecular weight marker. Lane 2 – Crude Lysate for A. rhizogenes expression. Lane 3 and 4 purified fractions of A. rhizogenes. (TIF) [file pone.0332702.s003.tif]

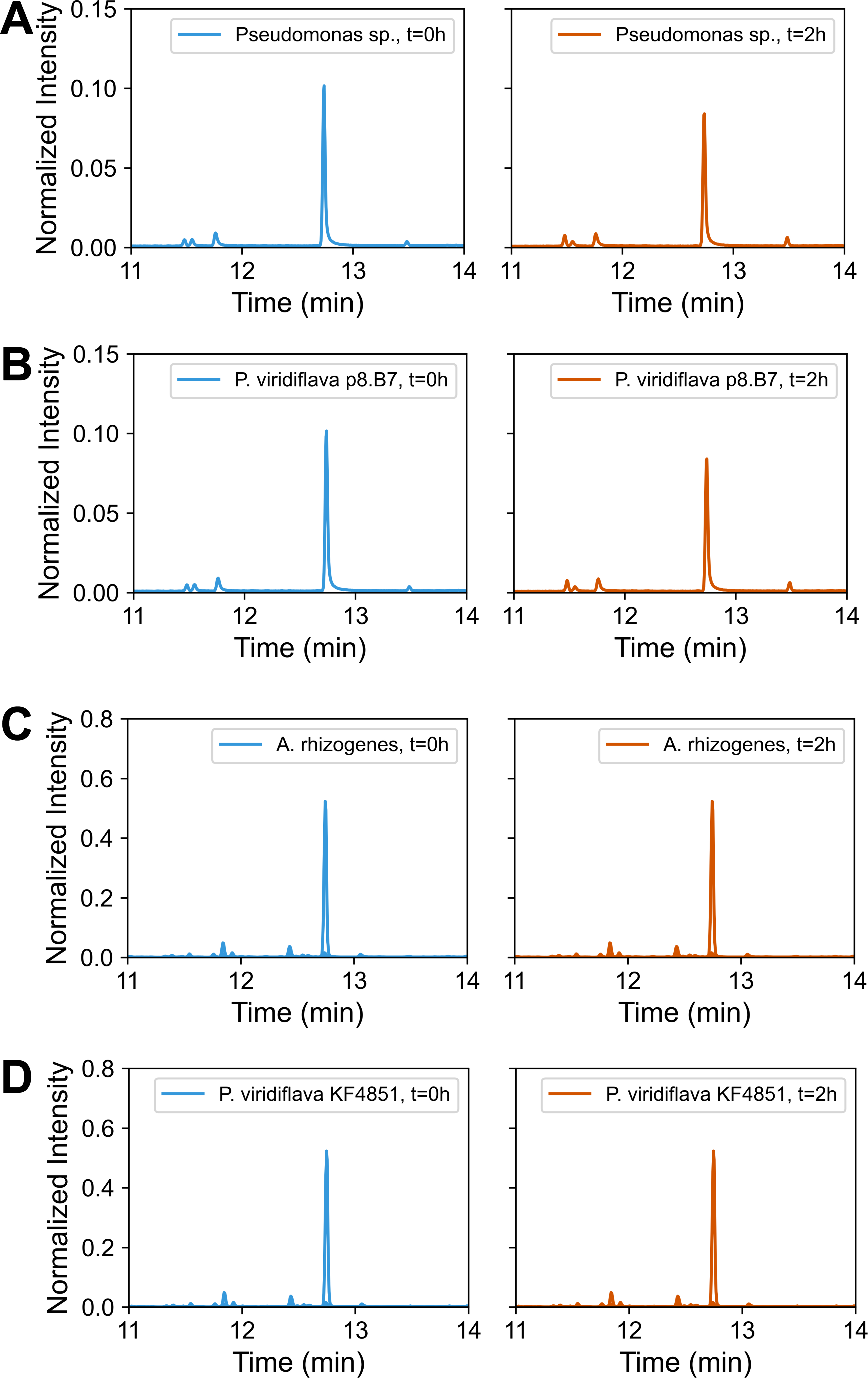

Supplement: S4 Fig — Assayed reaction conditions are as follows: (A) Wild-type N-Strep-tag® LadA homolog from Pseudomonas sp. ANT H4 (WP_149412683.1) (18 μM), wild-type N-Strep-tag® Fre (6 μM), hexadecane (1 mM, tR = 12.74 min), FMN (1 mM), NADH (1 mM) and HITENOL-AR 0.5% (m/v) for 2 hours at 37°C. (B) Wild-type N-Strep-tag® LadA homolog from P. viridiflava p8B7 (WP_122428611.1) (18 μM), wild-type N-strepTag Fre (6 μM), hexadecane (1 mM, tR = 12.74 min), FMN (1 mM), NADH (1 mM) and HITENOL-AR 0.5% (m/v) for 2 hours at 37°C. (C) Wild-type N-Strep-tag® LadA homolog from P. A. rhizogenes AF44 96 (WP_174015212.1) (18 μM), wild-type N-Strep-tag® Fre (6 μM), hexadecane (1 mM, tR = 12.74 min), FMN (1 mM), NADH (1 mM) and HITENOL-AR 0.5% (m/v) for 2 hours at 37°C. (D) Wild-type N-Strep-tag® LadA homolog from P. viridiflava KF485.1 (WP_088236120.1) (18 μM), wild-type N-Strep-tag® Fre (6 μM), hexadecane (1 mM, tR = 12.74 min), FMN (1 mM), NADH (1 mM) and HITENOL-AR 0.5% (m/v) for 2 hours at 37°C. Additional peak at tR = 11.54 corresponds to pentadecane (internal standard). (TIF) [file pone.0332702.s004.tif]

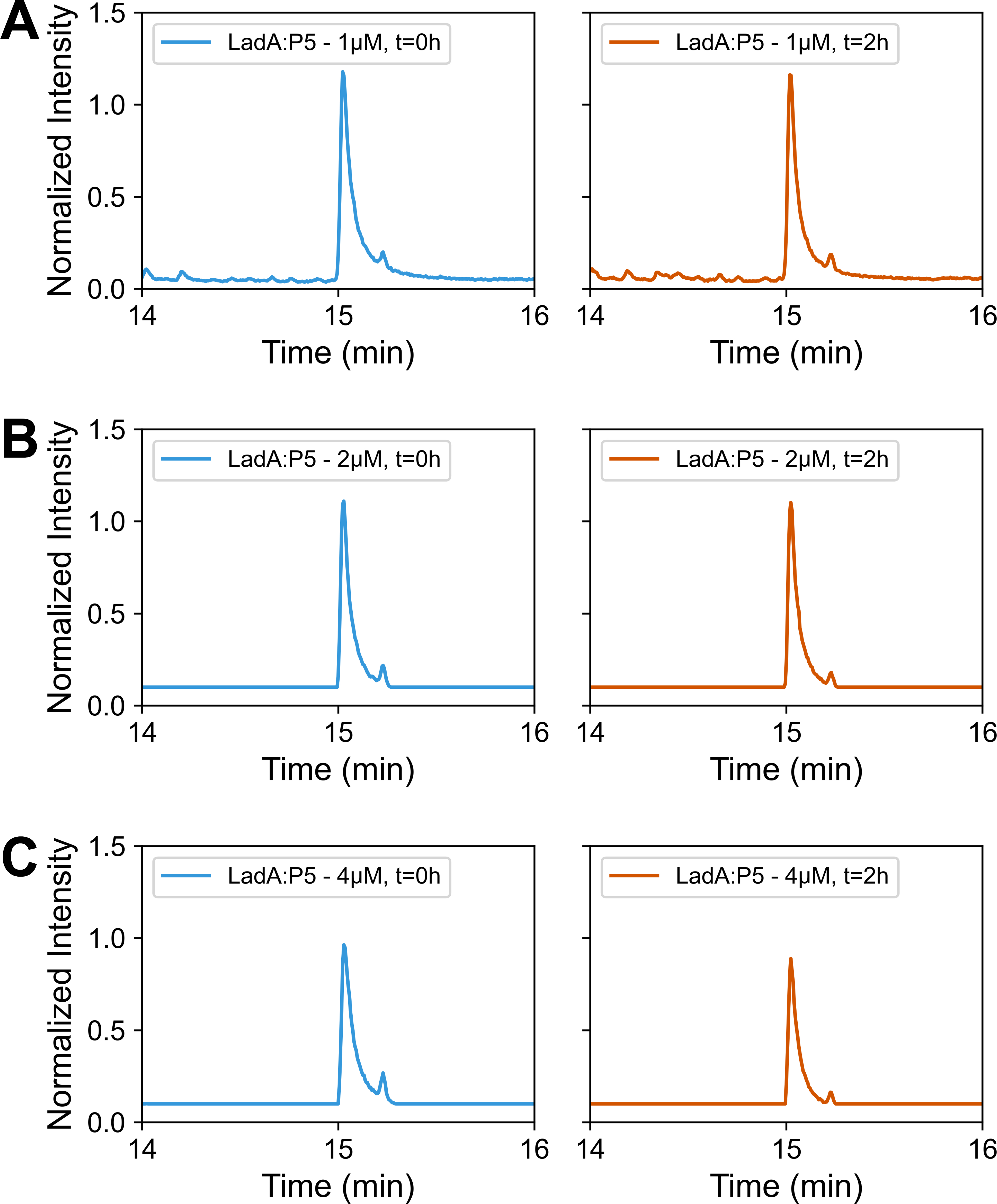

Supplement: S5 Fig — Assayed reaction conditions are as follows: (A) N-Strep-tag® LadA:P5 (1 μM), wild-type N-Strep-tag® Fre (1 μM), hexadecanone (1 mM, tR = 15.1 min), FMN (1 mM), NADH (1 mM) and HITENOL-AR 0.5% (m/v) for 2 hours at 37°C. (B) N-Strep-tag® LadA:P5 (2 μM), wild-type N-Strep-tag® Fre (1 μM), hexadecanone (1 mM, tR = 15.1 min), FMN (1 mM), NADH (1 mM) and HITENOL-AR 0.5% (m/v) for 2 hours at 37°C. (C) N-Strep-tag® LadA:P5 (5 μM), wild-type N-Strep-tag® Fre (1 μM), hexadecanone (1 mM, tR = 15.1 min), FMN (1 mM), NADH (1 mM) and HITENOL-AR 0.5% (m/v) for 2 hours at 37°C. (TIF) [file pone.0332702.s005.tif]

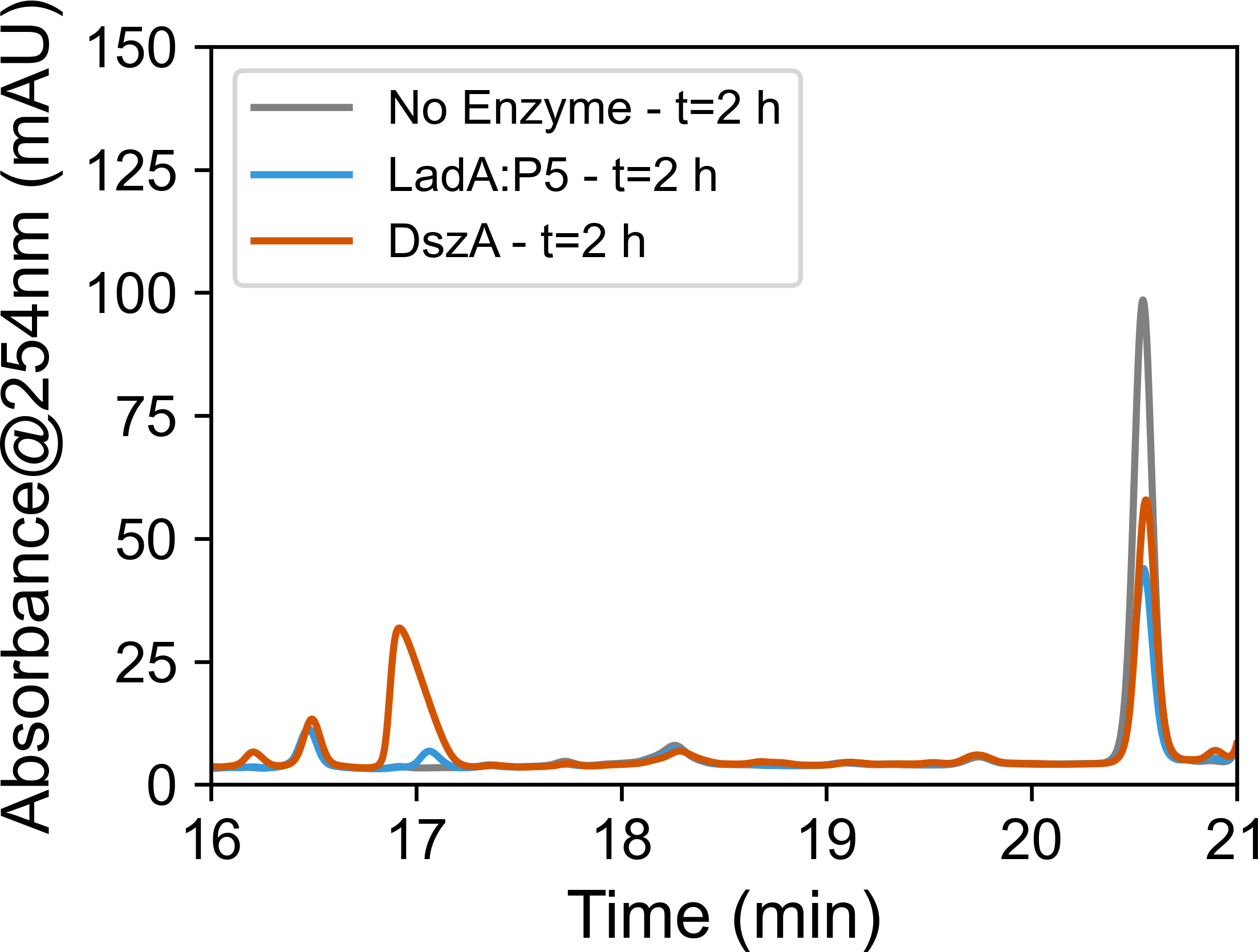

Supplement: S6 Fig — Assayed reaction conditions are as follows: N-Strep-tag® LadA:P5 (50 μM) or N-Strep-tag® DszA, wild-type N-Strep-tag® Fre (0.2 μM), dibenzothiophene sulfone (0.5 mM, tR = 20.5 min), FMN (1 mM), NADH (1 mM) for 2 hours at 37°C. (TIF) [file pone.0332702.s006.tif]
