# Supplementary material for: Can flavoprotein monooxygenases functionalize long-chain n-alkanes?
Source: PLoS One. 2025 Sep 19;20(9):e0332702. doi: 10.1371/journal.pone.0332702 (PMC12449030; doi:10.1371/journal.pone.0332702)
Supplement: S1 Table — (PDF) [file pone.0332702.s007.pdf]

Can flavoprotein monooxygenases functionalize long-chain *n*-alkanes?

Supporting Information

S1 Table. LadA PROSS-optimized variants.

Variant	Sequence
WT LadA	MGSWSHPQFEKGATKKIHINAFEMNCVGHIAHGLWRHPENQRHRYTDLNYWT<br>ELAQLLEKGKFDALFLADVVGIYDVYRQSRDTAVREAVQIPVNDPLMLISAMAY<br>VTKHLAFAVTFSTTYEHPYGHARRMSTLDHLTKGRIAWNVVTSHLPSADKNFGI<br>KKILEHDERYDLADEYLEVCYKLWEGSWEDNAVIRDIENNIYTDPSKVHEINHS<br>GKYFEVPGPHLCEPSPQRTPVIYQAGMSERGREFAAKHAECVFLGGKDVETL<br>KFFVDDIRKRAKKYGRNPDHIKMFAGICVIVGKTHDEAMEKLNSFQKYWSLEG<br>HLAHYGGGTGYDLSKYSSNDYIGSISVGEIINNMSKLDGKWFKLSVGTPKKVAD<br>EMQYLVEEAGIDGFNLVQYVSPGTFVDFIELVVPELQKRGLYRVDYEEGTYREK<br>LFGKGNYRLPDDHIAARYRNISSNV
LadA:P2	MGSWSHPQFEKGATKKIHINAFEMNCVGHIAHGLWRHPENQRHRYTDLNYWT<br>ELAQLLEKGKFDALFLADVVGIYDVYRQSRDTAVREAVQIPVNDPLMLISAMAY<br>VTKHLAFAVTFSTTYEHPYGFARRMSTLDHLTKGRIAWNVVTSHLPSADKNFGI<br>KKILEHDERYDLADEYLEVCYKLWEGSWEDDAVIRDRENNIYTDPSKVHEINHS<br>GKYFEVPGPHLCEPSPQRTPVIYQAGMSERGREFAAKHAECVFLGGKDVETL<br>KFFVDDIRKRAKKYGRNPDHIKMFAGICVIVGKTHDEAQEKLNSYQKYWSLEG<br>HLAHYGGGTGYDLSKYSSNDYIGSISVGEIINNMSKLDGKWFKLSVGTPKQVA<br>DEMQYWVEEAGIDGFNLVQYVSPGTFVDFIELVVPELQKRGLYRTDYEEGTYR<br>EKLFGKGNYRLPDDHIAARYRNISSNV
LadA:P5	MGSWSHPQFEKGATKKIHLNAFEMNCVGHIAHGLWRHPENQRHRYTDLNYW<br>TELAQLLEKGKFDALFLADVVGIYDVYRQSRDTAVREAVQIPVNDPLMLISAMA<br>AVTKHLGFAVTFSTTYEHPYTFARRMSTLDHLTKGRIAWNVVTSHLPSADKNFG<br>IKKILEHDERYDLADEYLEVCYKLWEGSWEDDAVIRDRENNIYTDPSKVHEINH<br>SGKYFEVPGPHLCEPSPQRTPVIYQAGMSERGREFAAKHAECVFLGGKDVET<br>LKFFVDDIRERAKKYGRNPDHIKFFAGICVIVGKTEEEAREKLEEYQKYWSLEG<br>HLAHYGGGTGYDLSKYSSNDYIGSISVGEIINNMSKLDGKWFKLSVGTPKQVA<br>DEMQYWVEEAGIDGFNLVQYVSPGTFVDFIELVVPELQKRGLYRTDYEEGTYR<br>EKLFGKGNYRLPDDHIAARYRNISSNV
DszA	MGAWSHPQFEKGATQQRQMHLAGFFSAGNVTHAHGAWRHTDASNDFLSGK<br>YYQHIARTLERGKFDLLFLPDGLAVEDSYGDNLDTGVGLGGQGAVALEPASVV<br>ATMAAVTEHLGLGATISATYYPPYHVARVFATLDQLSGGRVSWNVVTSLNDAEA<br>RNFGINQHLEHDARYDRADEFLEAVKKLWNSWDEDALVLDKAAGVFADPAKV<br>HYVDHHGEWLNVRGPLQVPRSPQGEPVILQAGLSPRGRRFAGKWAEAVFSLA<br>PNLEVMQATYQGIKAEVDAAGRDPDQTKIFTAVMPVLGESQAVAQERLEYLNS<br>LVHPEVGLSTLSSHTGINLAAYPLDTPIKDILRDLQDRNVPTQLHMFAAATHSEE<br>LTLAEMGRRYGTNVGFVPQWAGTGEQIADELIRHFEGGAADGFIISPAFLPGSY<br>DEFVDQVVPVLQDRGYFRTEYQGNTLRDHLGLRVPQLQGQPS
