# Supplementary material for: Can flavoprotein monooxygenases functionalize long-chain n-alkanes?
Source: PLoS One. 2025 Sep 19;20(9):e0332702. doi: 10.1371/journal.pone.0332702 (PMC12449030; doi:10.1371/journal.pone.0332702)
Supplement: S2 Table — (PDF) [file pone.0332702.s008.pdf]

Can flavoprotein monooxygenases functionalize long-chain *n*-alkanes?

Supporting Information

S2 Table. LadA queries and homologous sequences.

**Clade color**	**Organism**	**Assembly ID**	**RefSeq ID**
N/A	*Geobacillus thermodenitrificans* NG80-2	GCA_000015745.1	WP_011888513.1
N/A	*Geobacillus thermoleovorans* B23	GCA_000474195.1	WP_014196861.1
N/A	*Geobacillus thermoleovorans* B23	GCA_000474195.1	WP_014196855.1
N/A	*Geobacillus thermoleovorans* B23	GCA_000474195.1	WP_014196856.1
Black	*Pseudomonas* sp. ANT H4	GCA_008369295.1	WP_149412683.1
Blue	*Pseudomonas viridiflava* isolate p8.B7	GCA_900601525.1	WP_122428611.1
Cyan	*Streptomyces erythrochromogenes* NRRL B-2112	GCA_000725555.1	WP_031151001.1
Yellow	*Burkholderia* sp. AU33423	GCA_002223135.1	WP_089449490.1
Orange	*Pseudomonas poae* MYb117	GCA_002980135.1	WP_105696424.1
Purple	*Mesorhizobium* sp.	GCA_000502355.1	WP_023806228.1
Red	*Agrobacterium rhizogenes* AF44 96	GCA_013322085.1	WP_174015212.1
Magenta	*Pseudomonas viridiflava* KF485 1	GCA_019083885.1	WP_088236120.1
