# Supplementary material for: Can flavoprotein monooxygenases functionalize long-chain n-alkanes?
Source: PLoS One. 2025 Sep 19;20(9):e0332702. doi: 10.1371/journal.pone.0332702 (PMC12449030; doi:10.1371/journal.pone.0332702)
Supplement: S3 Table — (PDF) [file pone.0332702.s009.pdf]

Can flavoprotein monooxygenases functionalize long-chain *n*-alkanes?

Supporting Information

S3 Table. Primers used for cloning Fre into NStrepTag-pET-28(a+) vector.

Primer name	5’ to 3’ sequence
NStrep_Fre_F	CTCAGTTTGAGAAAGGTGCAACAACCTTAAGCTGTAAAGTGACCTCGGTA
NStrep_Fre_R	TTTAGCAGCCTAGGTATTAATCAGATAAATGCAAACGCATCGCCAAACAG
pET-28_F	TTAATACCTAGGCTGCTAAACAAAGCCCGAAAGG
pET-28_R	TGCACCTTTCTCAAACTGAGGATGACTCCA
