# Supplementary material for: Can flavoprotein monooxygenases functionalize long-chain n-alkanes?
Source: PLoS One. 2025 Sep 19;20(9):e0332702. doi: 10.1371/journal.pone.0332702 (PMC12449030; doi:10.1371/journal.pone.0332702)
Supplement: S1 Raw data — (PDF) [file pone.0332702.s010.pdf]

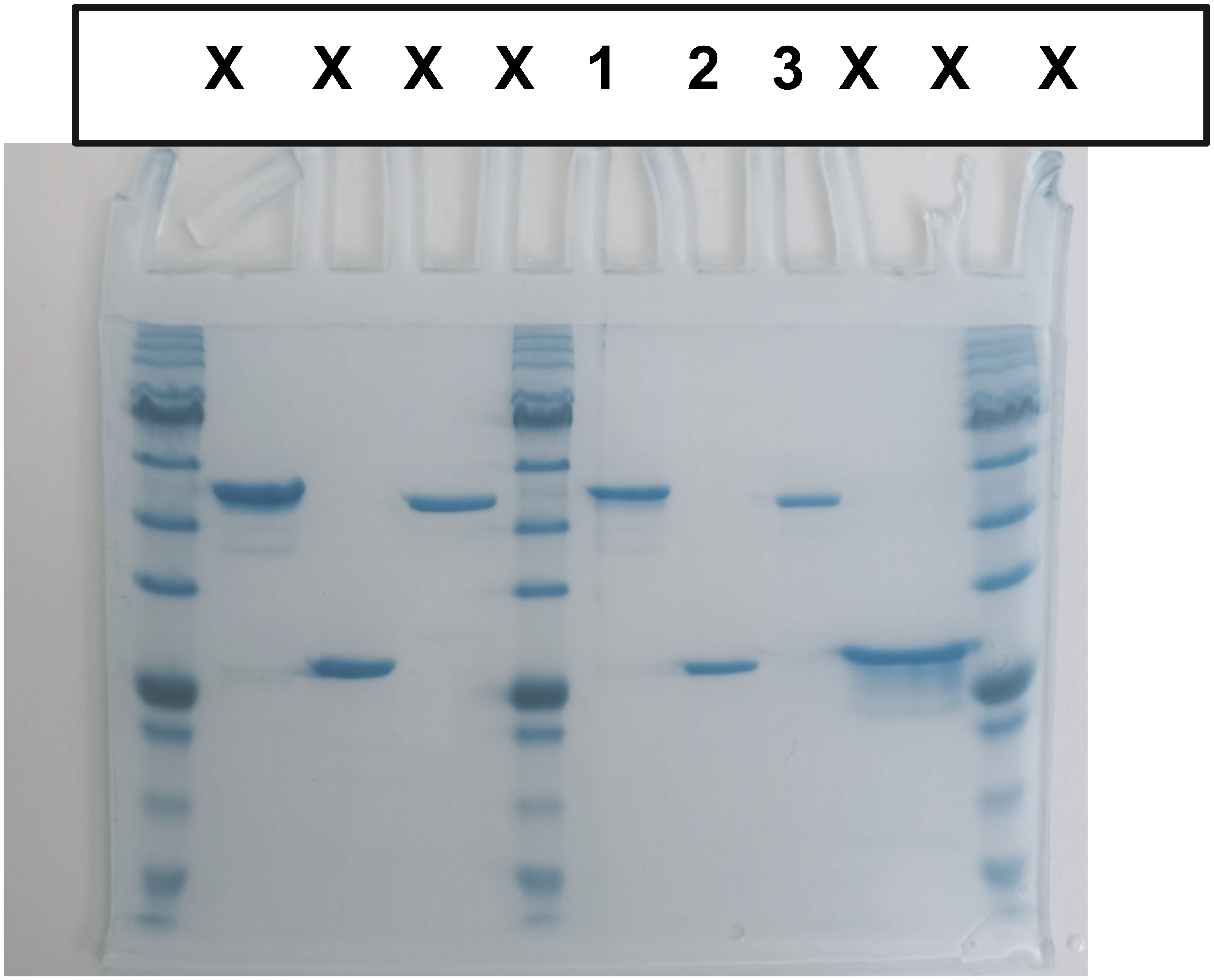

SDS-PAGE in Figure 1.

Lane 1: Molecular weight (MW) marker.

Lane 2: purified *wild-type* N-strepTag LadA

Lane 3: purified *wild-type* N-strepTag Fre

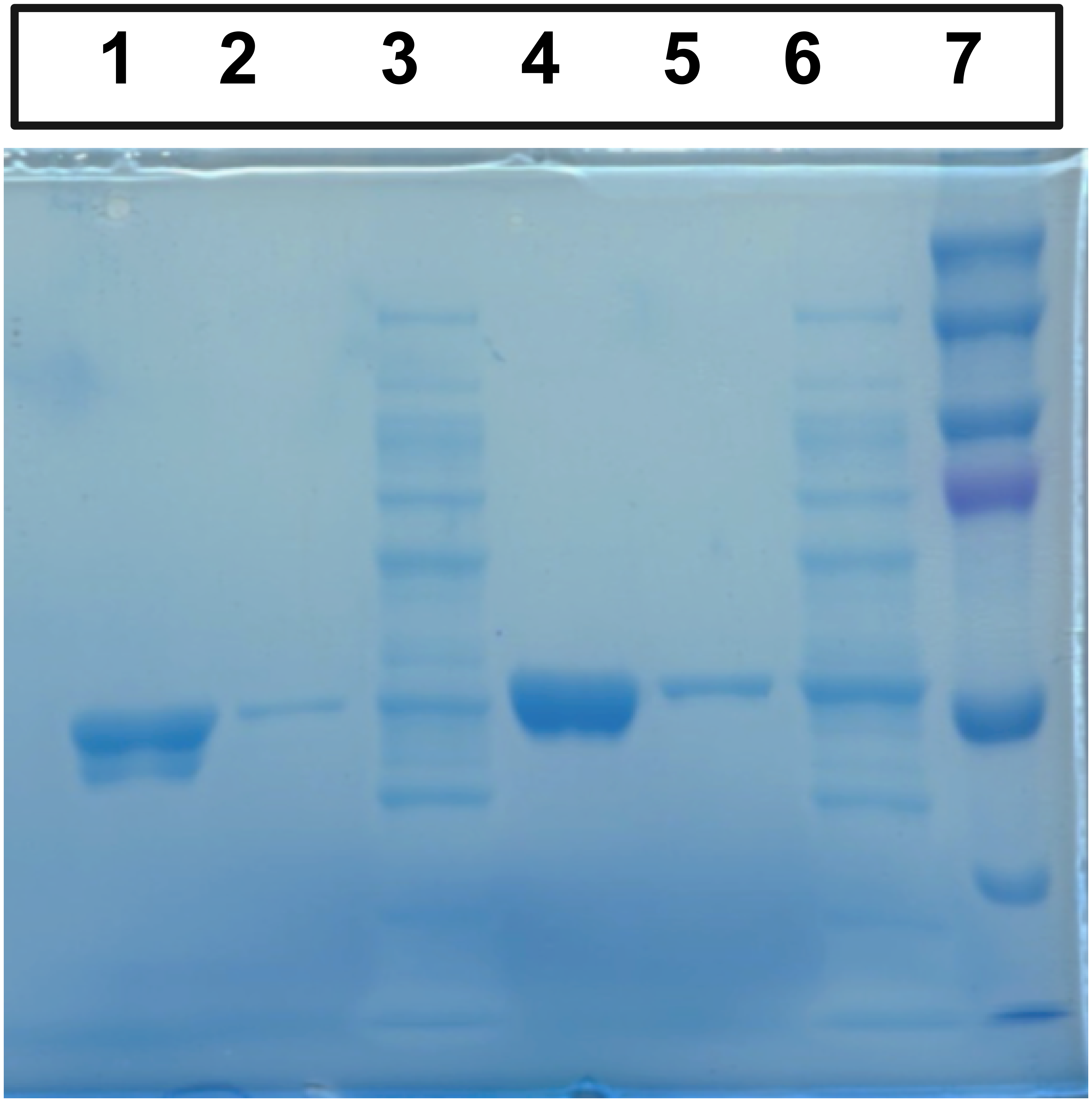

SDS-PAGE in Figure S3A.

Lane 1 and 2 purified fractions of *Pseudomonas* sp. ANT H4*.*

Lane 3 - Crude Lysate for *Pseudomonas* sp. ANT H4.

Lane 4 and 5 - purified fractions of *P. viridiflava* p8B7.

Lane 6 - Crude Lysate for *P. viridiflava* p8B7*.*

Lane 7 - Molecular weight marker

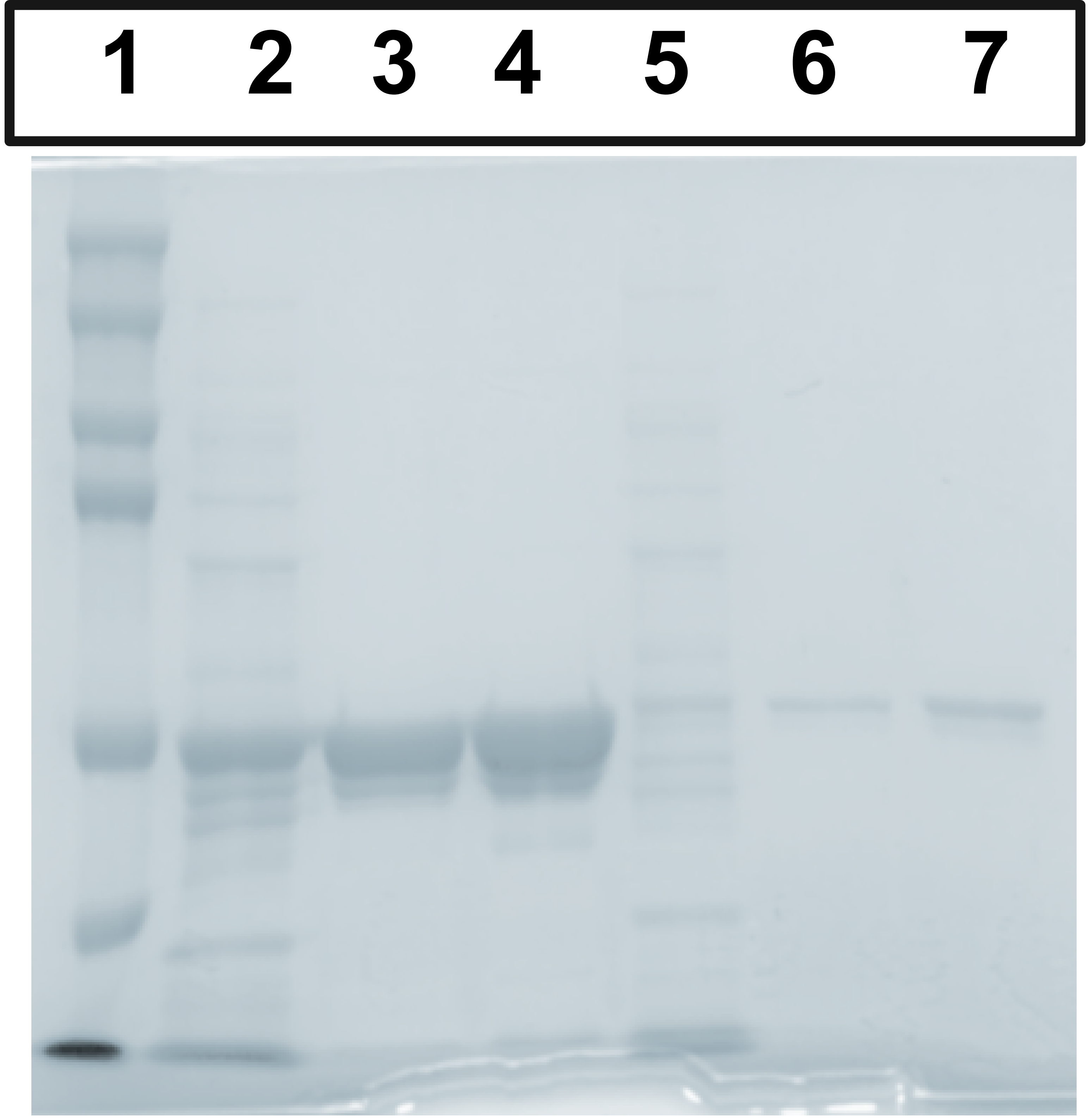

SDS-PAGE for Figure S3B.

Lane 1 - Molecular weight marker.

Lane 2 - Crude Lysate for *Mesorhizobium* sp. production

Lane 3 and 4 - purified fractions of *Mesorhizobium* sp.

Lane 5 - Crude Lysate for *P. viridiflava* KF4851

Lane 6 and 7 - purified fractions of *P. viridiflava* KF4851

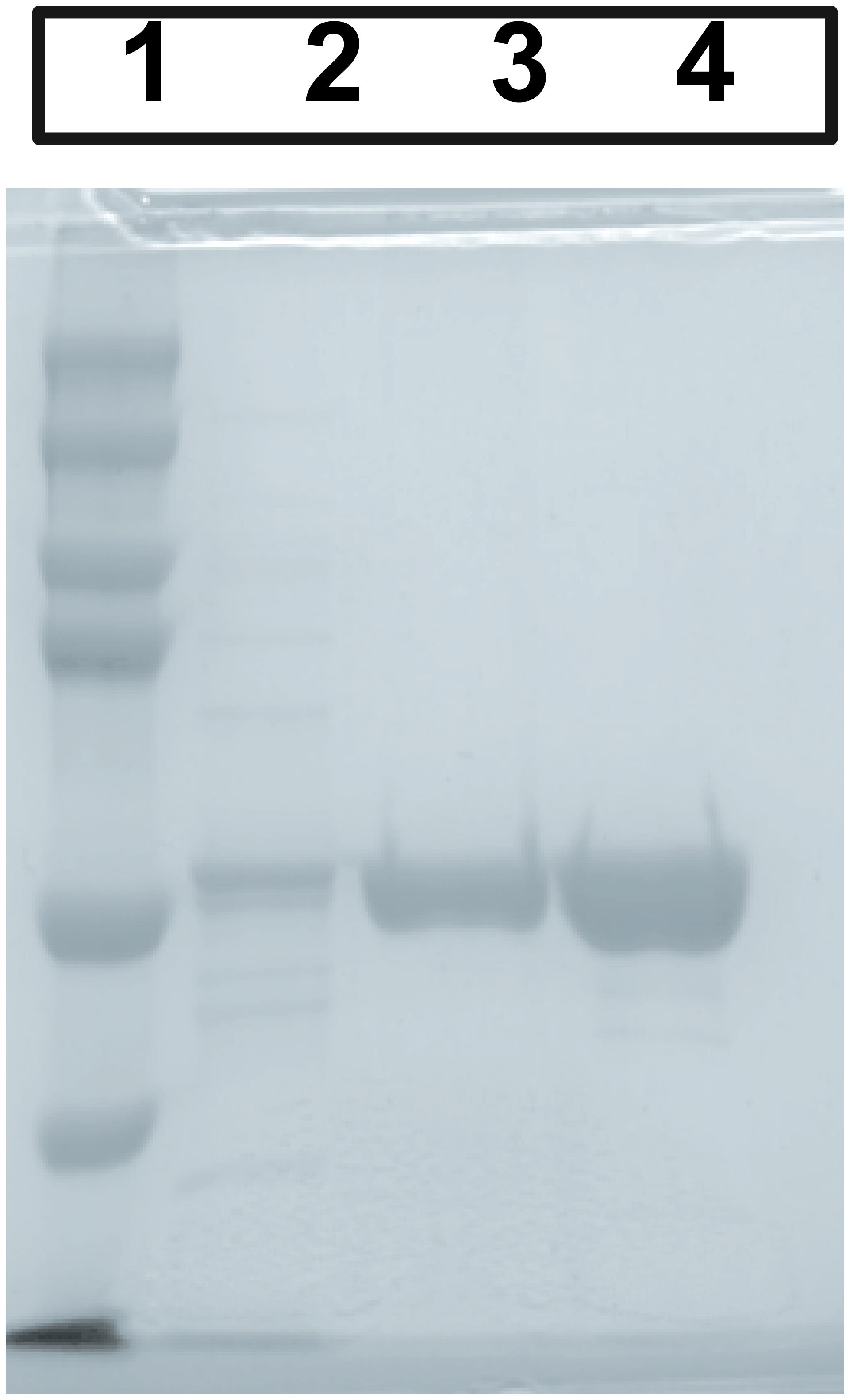

SDS-PAGE for Figure S3C.

Lane 1 - Molecular weight marker.

Lane 2 - Crude Lysate for *A. rhizogenes* production .

Lane 3 and 4 - purified fractions of *A. rhizogenes.*
